# A Screening Tool to Predict Sepsis in Patients With Suspected Infection in the Emergency Department

**DOI:** 10.7759/cureus.78728

**Published:** 2025-02-08

**Authors:** Yasufumi Oi, Fumihiro Ogawa, Hiroshi Honzawa, Takeru Abe, Shouhei Imaki, Ichiro Takeuchi

**Affiliations:** 1 Emergency Care Department, Yokohama City University Hospital, Yokohama, JPN; 2 Center for Integrated Science and Humanities, Fukushima Medical University, Fukushima, JPN; 3 Emergency and Critical Care Medical Center, Yokohama Municipal Citizen’s Hospital, Yokohama, JPN; 4 Advanced Critical Care and Emergency Center, Yokohama City University Hospital, Yokohama, JPN

**Keywords:** lactate level, modified early warning score, national early warning score, quick sequential organ failure assessment, sepsis, systemic inflammatory response syndrome score

## Abstract

Background and objective

Sepsis is a life-threatening condition associated with high morbidity and mortality, and hence early recognition and treatment are crucial. The 2016 Sepsis-3 guidelines introduced the quick Sequential Organ Failure Assessment (qSOFA), but its low sensitivity limits early detection. The 2021 Surviving Sepsis Campaign Guidelines (SSCG) discourage relying solely on qSOFA and recommend additional tools such as the systemic inflammatory response syndrome (SIRS) score, the National Early Warning Score (NEWS), and the Modified Early Warning Score (MEWS) along with lactate measurement. This study assessed whether combining qSOFA with quantitative capillary refill time (Q-CRT) or lactate levels enhances early sepsis diagnosis in emergency departments.

Methods

This retrospective, multi-facility observational study was conducted at two hospitals in Yokohama, Japan. Patients with suspected infections who underwent Q-CRT measurement were included. Q-CRT was measured using a pulse oximeter-based device that records the time taken for blood flow to return to 90% after compression. Receiver operating characteristic (ROC) curves determined the area under the curve (AUC), sensitivity, and specificity. Statistical significance was set at p<0.05.

Results

Of the 357 patients who underwent Q-CRT measurement, 75 (21%) were suspected of having an infection, with 48 (64%) classified as having sepsis with organ dysfunction. Patients in the sepsis group had higher age, heart rate, lactate level, creatinine level, NEWS, MEWS, and Sequential Organ Failure Assessment (SOFA) scores compared to those without organ dysfunction. Among individual tools, the qSOFA, NEWS, and MEWS scores showed high AUCs (>0.8), while Q-CRT and lactate levels demonstrated moderate predictive accuracy with AUCs exceeding 0.7. The SIRS score had the lowest predictive ability, with an AUC of approximately 0.6. Combining qSOFA with Q-CRT or lactate levels significantly improved sensitivity and specificity. The qSOFA+Q-CRT combination resulted in an AUC of 0.821, sensitivity of 83.3%, and specificity of 81.4%, while the qSOFA+lactate combination yielded an AUC of 0.844, sensitivity of 87.5%, and specificity of 81.4%. These combinations exceeded 80% in both sensitivity and specificity, unlike the SIRS-based combinations, which showed limited improvement and specificity below 40%. While the qSOFA score alone demonstrated limited sensitivity, combining it with Q-CRT or lactate levels enhanced its predictive performance for early sepsis detection. This approach improved sensitivity without compromising specificity. The increase in sensitivity and specificity is likely due to Q-CRT and lactate identifying sepsis cases not detected by qSOFA, thereby making the combined approach more reliable for clinical use. Lactate levels are well-established markers associated with sepsis severity, and Q-CRT offers a non-invasive means of assessing peripheral perfusion.

Conclusions

Combining qSOFA with Q-CRT or lactate levels significantly improves early sepsis detection by enhancing both sensitivity and specificity. These combinations offer superior diagnostic accuracy compared to standalone tools, supporting their potential integration into clinical protocols for better patient outcomes. Further prospective studies are needed to validate these findings across diverse clinical settings.

## Introduction

Sepsis is a life-threatening organ dysfunction caused by a dysregulated host response to an infection [1]. It affects millions of people worldwide annually, with a mortality rate of one in three to six, posing a significant medical challenge [2-4]. Rapid recognition and management within the first few hours are crucial for improving outcomes. Therefore, early identification of sepsis is vital. Various screening tools have been developed for this purpose, including the systemic inflammatory response syndrome (SIRS) score, the quick Sequential Organ Failure Assessment (qSOFA) score, the Sequential Organ Failure Assessment (SOFA) score, the National Early Warning Score (NEWS), and the Modified Early Warning Score (MEWS), and markers such as vital signs and signs of infection have been reported [5,6].　

The 2016 Sepsis-3 guidelines recommend the diagnosis of sepsis using the qSOFA score, a new screening tool. The qSOFA score comprises three criteria: a Glasgow Coma Scale (GCS) score <15, respiratory rate ≥22 breaths/min, and systolic blood pressure ≤100 mmHg. If two or more criteria are met, the qSOFA result is considered positive, predicting an increased mortality risk and prolonged ICU stay in patients with suspected sepsis [7]. However, using the qSOFA score as a screening tool is not endorsed by other studies [8,9], although it has been identified as a predictor of poor outcomes in patients with suspected infections. Studies investigating the potential of the qSOFA score as a sepsis screening tool have indicated that it has a lower sensitivity than the SIRS score, NEWS, and MEWS [10].

The 2021 Surviving Sepsis Campaign Guidelines (SSCG) advise against using only the qSOFA score for sepsis or septic shock screening; instead, they recommend using the SIRS score, NEWS, or MEWS [11]. Owing to the low sensitivity of the qSOFA score, the guidelines emphasize the importance of early identification of organ dysfunction and recommend against using only the qSOFA score for screening, endorsing lactate level measurement.

To increase the predictive capabilities of new sepsis screening indicators, we developed the quantitative capillary refill time (Q-CRT), qSOFA score, and SIRS score combination, which were compared in the emergency department. The capillary refill time, a noninvasive sign of shock, is subjective because it involves human-assessed measurements. The Q-CRT, developed for objective measurement, reportedly correlates with lactate levels [12]. Studies have shown that combining the Q-CRT with the qSOFA score results in a higher sensitivity and specificity than using only the qSOFA score [13]. As an early sepsis diagnosis significantly affects prognosis, this study aimed to explore whether combining screening tools could improve early diagnostic accuracy.

## Materials and methods

Study setting and ethical approval

This retrospective, two-facility observational study was conducted at Yokohama City University Hospital and Yokohama Municipal Citizen’s Hospital in Yokohama, Japan. Yokohama City University Hospital serves the southern region, whereas Yokohama Municipal Citizen’s Hospital covers the central region, with a combined population of approximately 3.7 million in 2018. The study was approved by the Ethics Committee of Yokohama Municipal Citizen’s Hospital (approval numbers 17-07-01), and informed consent was obtained from all patients or their families.

Aim and design

In this retrospective observational study, we investigated the association between Q-CRTs and sepsis in patients in the emergency department. Previous studies have validated the utility of the qSOFA score, NEWS, MEWS, and SIRS score in predicting sepsis [1]. However, the predictive accuracy of combining these with the Q-CRT and lactate levels, which may enhance prediction speed and accuracy, remains to be determined. Therefore, we first evaluated the predictive accuracy of the qSOFA score, NEWS, MEWS, and SIRS score and then assessed the Q-CRT and lactate levels. Additionally, we compared the predictive accuracy of eight combinations of the four existing tools with either the Q-CRT or lactate levels.

Patients

Patients suspected of having an infection whose Q-CRTs were measured in the emergency department and transported by ambulance were included. Emergency physicians exclusively performed Q-CRT measurements, particularly during the initial evaluation upon emergency admission for patients suspected of having infections. Q-CRT was measured immediately after arrival at the emergency department. A suspected infection was defined as any culture performed within 72 hours of intravenous antibiotic administration or the administration of intravenous antimicrobial agents within 24 hours of a culture being performed [1].

We excluded patients with physical conditions preventing Q-CRT measurement, such as finger injuries, and patients on dialysis due to the presence of shunts in their arms who could not receive intravenous infusions. Only one device was used to measure the Q-CRTs, and all measurements were performed by two emergency physicians.

Lactate levels were assessed using arterial blood gas analysis obtained during the initial evaluation upon emergency admission. All samples were obtained during the initial evaluation upon emergency admission. In cases of respiratory failure, lactate levels were measured concurrently with arterial blood analysis, while in cases of suspected infection, they were measured at the time of blood culture collection. The median time from arrival to blood gas analysis was 10 minutes [interquartile range (IQR): 8-13 minutes].

Measurement of Q-CRT

**Figure 1:**
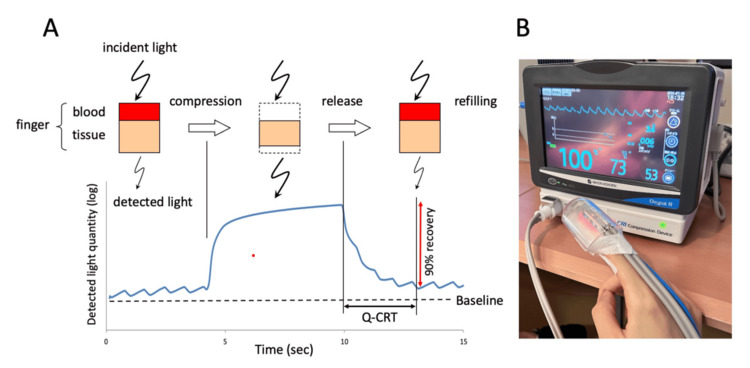
Schematic representation (A) and photo (B) of Q-CRT measurement A: The quantity of detected light measured by a pulse oximeter equipped with a sensor for oxygen saturation measurement is related to the blood volume, based on the Lambert-Beer law. B: Diagram of a compression device mounted on a saturation monitor Q-CRT: quantitative capillary refill time

The Q-CRT was measured using a device equipped with an oxygen saturation (SpO_2_) sensor (Figure 1) [12]. The amount of absorbed light, measured using a pulse oximeter based on the Lambert-Beer law, is related to the blood volume. The patient's index finger was compressed with a mechanical pressure of 500 mmHg for five seconds, stopping the blood flow and increasing the amount of detected light. Blood flow resumed once the pressure was released, and the detected light decreased. The time in seconds from the pressure release until the blood flow reached 90% of its original value was defined as the Q-CRT.

Definition of qSOFA score, SIRS score, and sepsis

The qSOFA score ranges from 0 to 3, with a systolic blood pressure ≤100 mmHg, respiratory rate ≥22 breaths/minute, and altered mental status receiving 1 point each. A score of ≥2 indicates suspected sepsis. The SIRS score includes the following criteria: heart rate >90 beats/minute, respiratory rate >20 breaths/minute or PaCO_2_ <32 mmHg, white blood cell count <4000 or >12000 cells/mm³, and temperature <36 °C or ≥38 °C. Sepsis is considered to be present when at least two of the criteria are met. Sepsis, as defined using the Sepsis-3 guidelines, is a life-threatening organ dysfunction caused by a dysregulated response to an infection. Organ dysfunction is indicated by an increase in the SOFA score by 2 or more points.

Definition of NEWS and MEWS

The UK National Health Service uses the NEWS, where a higher score indicates a greater risk of patient deterioration [14]. The NEWS includes seven items: respiratory rate, SpO_2_, oxygen administration, temperature, systolic blood pressure, heart rate, and consciousness, with 0 to 3 points per item and a maximum score of 20 (Table 1). A score of ≥7 indicates a high risk, requiring an emergency response or ICU transfer. The MEWS has a maximum of 14 points, with 0-3 points each assigned for respiratory rate, heart rate, systolic blood pressure, level of consciousness, and temperature [15]. A score of ≥7 activates the Rapid Response System [16].

**Table 1:** Definisitons of NEWS and MEWS AVPU: alert/verbal/pain/unresponsive (V: responds to verbal stimuli, P: responds to painful stimuli, U: unresponsive); MEWS: Modified Early Warning Score; NEWS: National Early Warning Score; SpO_2_: oxygen saturation

Physiological parameters (score)		3	2	1	0	1	2	3
National Early Warning Score (NEWS)								
	Respiration rate (breaths per minute)	≤8	ー	9–11	12–20	ー	21–24	≥25
	SpO_2 _(%)	≤91	92–93	94–95	≥96	ー	ー	ー
	Any supplemental oxygen	ー	Yes	ー	No	ー	ー	ー
	Temperature (℃)	≤35.0	ー	35.1–36.0	36.1–38.0	38.1–39.0	≥39.1	ー
	Systolic blood pressure (mmHg)	≤90	91–100	101–110	111–219	ー	ー	≥220
	Heart rate (beats per minute)	≤40	ー	41–50	51–90	91–110	111–130	≥131
	Level of consciousness assessed using the AVPU system	ー	ー	ー	Alert	ー	ー	V, P, U
Modified Early Warning Score (MEWS)								
	Systolic blood pressure (mmHg)	≤70	71–80	81–100	101–199	ー	≥200	ー
	Heart rate (beats per minute)	ー	≤40	41–50	51–100	101–110	111–129	≥130
	Respiration rate (breaths per minute)	ー	≤8	ー	9–14	15–20	21–29	≥30
	Temperature (℃)	ー	≤35.0	ー	35.1–38.4	ー	≥38.5	ー
	Level of consciousness assessed using the AVPU system	ー	ー	ー	Alert	V	P	U

Data analysis and statistical methods

Data analysis was performed using Stata version 13.1. Continuous variables are presented as medians and IQR, and categorical variables are presented as counts and percentages. Univariate analysis was performed using Student's t-test, the Mann-Whitney U test, and the chi-squared test. The sensitivity, specificity, and area under the curve (AUC) were calculated for Q-CRTs, qSOFA scores ≥2, SIRS scores ≥2, NEWSs, MEWSs, heart rates, and lactate levels to assess their predictive ability for sepsis. DeLong's test was used to compare the AUCs. Statistical significance was set at p<0.05. The cutoff values for Q-CRT and lactate levels were determined based on the point with the highest sensitivity and specificity on the ROC curve. Since this analysis compares and examines the scores of each screening tool, a multivariable analysis considering confounding factors has not been performed.

## Results

**Figure 2:**
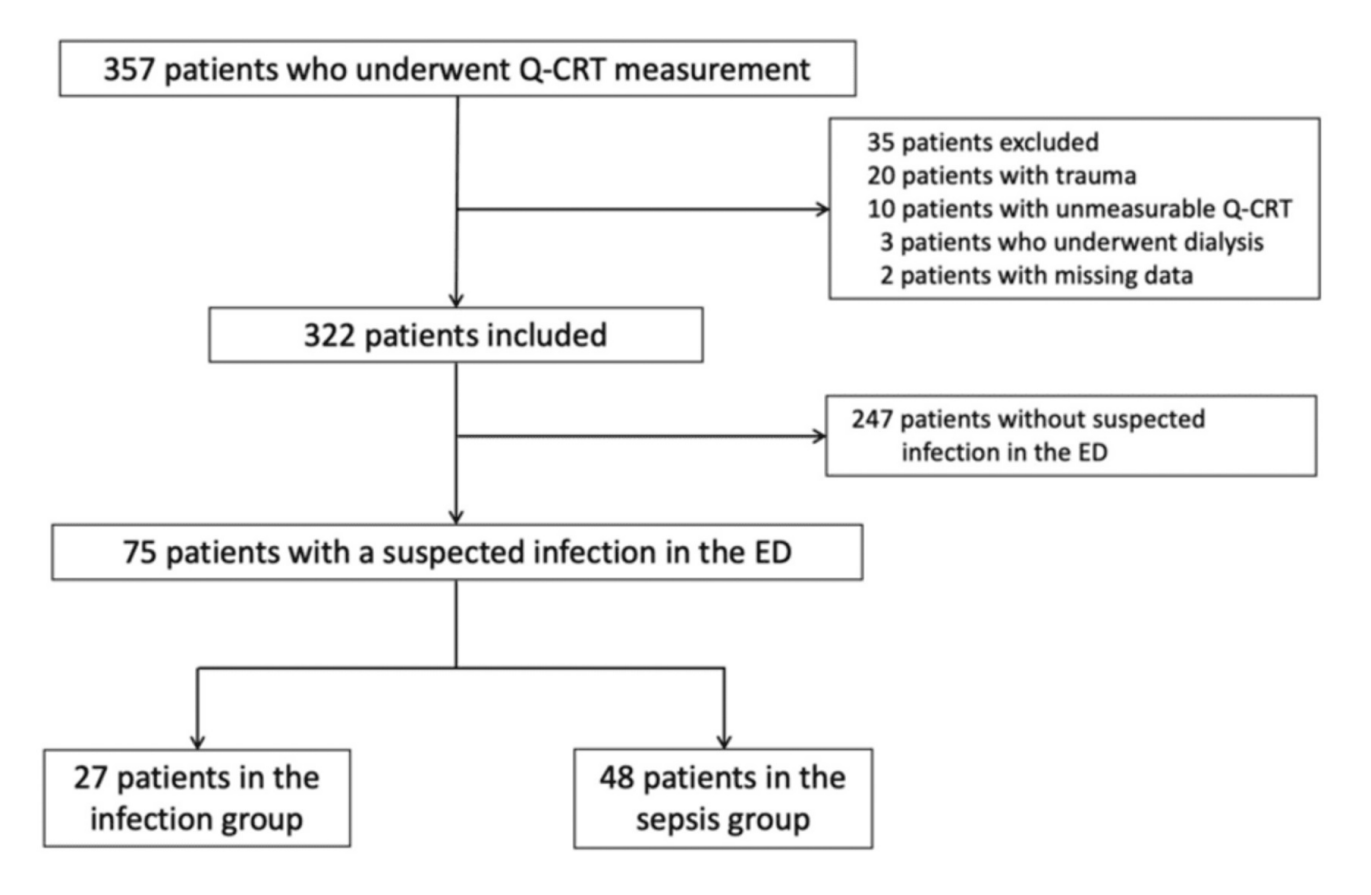
Study flow chart ED: emergency department; Q-CRT: quantitative capillary refill time

Among the 1,323 patients transported by ambulance to Yokohama City University Hospital between November 2015 and March 2017, the Q-CRT was measured in 286 patients (21.6%), and 31 were suspected of having an infection. At Yokohama Municipal Citizen’s Hospital, the Q-CRT was measured in 71 of 1,152 patients (6.1%) transported by ambulance between August 2017 and April 2018, and 44 were suspected of having an infection. During the study period, 75 patients (21%) were suspected of having an infection, including 27 (36%) in the infection group (infection without organ dysfunction) and 48 (64%) in the sepsis group (infection with organ dysfunction) (Figure 2). The comprehensive data on patients with infection are provided in the Appendices.

Comparison between the two groups

Patients in the sepsis group had a higher age, heart rate, creatinine level, lactate level, Q-CRT, NEWS, MEWS, and SOFA scores, and a lower SpO_2_ and platelet count than those in the infection group (Table 2). Additionally, a higher proportion of patients in the sepsis group had qSOFA and SIRS scores of ≥2 and GCS scores of <15 compared to those in the non-sepsis group.

**Table 2:** Comparison between the infection and sepsis groups Univariate analysis was performed using Student's t-test, the Mann–Whitney U test, and the chi-squared test. Statistical significance was set at p<0.05. BT: body temperature; GCS: Glasgow Coma Scale; HR: heart rate; MEWS: Modified Early Warning Score; NEWS: National Early Warning Score; Q-CRT: quantitative capillary refill time; qSOFA: quick Sequential Organ Failure Assessment; RR: respiratory rate; SBP: systolic blood pressure; SIRS: systemic inflammatory response syndrome; SpO_2_: oxygen saturation; WBC: white blood cells

Variable	Infection group (n=27)	Sepsis group (n=48)	P-value
Male sex, n (%)	19 (70.3)	32 (66.7)	0.741
Age, years, median (IQR)	69 (57–78)	81 (65.5–84.5)	0.016
SBP, mmHg, median (IQR)	132 (122–146)	129 (105–150)	0.54
RR, breaths/minute, median (IQR)	24 (20–26)	25 (18–30)	0.268
HR, beats/minute, median (IQR)	96 (84–108)	109.5 (96–128)	0.006
GCS score <15, n (%)	3 (11.1)	34 (70.8)	<0.001
BT, °C, median (IQR)	37.8 (36.7–38.4)	37.9 (37.1–39.2)	0.279
SpO_2_, %, median (IQR)	96 (94–98)	95 (91.5–97)	0.034
WBC, /μL, median (IQR)	12,510 (9,620–17,500)	1,1030 (8,595–13,325)	0.294
Creatinine level, mg/dL, median (IQR)	0.81 (0.73–0.92)	0.99 (0.77–1.62)	0.006
Bilirubin level, mg/dL, median (IQR)	0.7 (0.5–1.1)	1.05 (0.6–1.75)	0.067
Platelet count, 10^3^/μL, median (IQR)	21.4 (18.4–25.5)	18.4 (10.8–22.2)	0.009
Lactate level, mmol/L, median (IQR)	1.4 (1.1–1.6)	1.99 (1.51–3.07)	<0.001
Lactate level >1.7, n (%)	5 (18.5)	34 (70.8)	<0.001
SOFA score, median (IQR)	1 (0–1)	3.5 (2–5.5)	<0.001
qSOFA ≥2, median (IQR)	0	32 (66.7)	<0.001
SIRS ≥2, n (%)	16 (59.2)	39 (81.2)	0.039
NEWS, median (IQR)	4 (2–6)	9.5 (7–12)	<0.001
NEWS >7.5, n (%)	2 (7.4)	33 (68.7)	<0.001
MEWS, median (IQR)	3 (2–4)	6 (4.5–8)	<0.001
MEWS >4.5, n (%)	2 (7.4)	36 (75)	<0.001
Q-CRT, seconds, median (IQR)	2.207 (1.591–3.269)	3.923 (2.529–6.694)	<0.001
Q-CRT ＞3.5, n (%)	5 (18.5)	28 (58.3)	<0.001

Individual predictive ability of the screening tools for sepsis

The qSOFA score, NEWS, and MEWS exhibited AUCs >0.8. Q-CRTs and lactate levels displayed AUCs >0.7, whereas the AUC for the SIRS score was approximately 0.6. A significant difference in the predictive ability for sepsis was observed between the qSOFA and SIRS scores (AUC: 0.833 vs. 0.610) (Table 3).

**Table 3:** Individual predictive ability of the screening tools for sepsis The sensitivity, specificity, and AUC were calculated for Q-CRTs, qSOFA scores ≥2, SIRS scores ≥2, NEWSs, MEWSs, heart rates, and lactate levels to assess their predictive ability for sepsis. DeLong's test was used to compare the AUCs. Statistical significance was set at p<0.05. AUC: area under the curve; CI: confidence interval; MEWS: Modified Early Warning Score; NEWS: National Early Warning Score; Q-CRT: quantitative capillary refill time; qSOFA: quick Sequential Organ Failure Assessment; SIRS: systemic inflammatory response syndrome

	AUC (95% CI)	Sensitivity	Specificity	Difference between areas (95% CI)	P-value	cutoff value	Unit
qSOFA	0.833 (0.765–0.900)	66.7	100	ー	ー	ー	
SIRS	0.610 (0.500–0.719)	81.2	40.7	0.223 (0.105–0.342)	<0.001		
NEWS	0.846 (0.756–0.937)	69	93	0.0135 (–0.066–0.093)	0.739	7.5	
MEWS	0.846 (0.759–0.934)	75	93	0.0135 (-0.062–0.089)	0.728	4.5	
Q-CRT	0.740 (0.628–0.853)	59	81	0.0926 (-0.038–0.224)	0.166	3.5	Seconds
Lactate	0.769 (0.657–0.880)	73	81	0.064 (-0.057–0.185)	0.299	1.7	mmol/L

Combined predictive ability of the screening tools for sepsis

**Figure 3:**
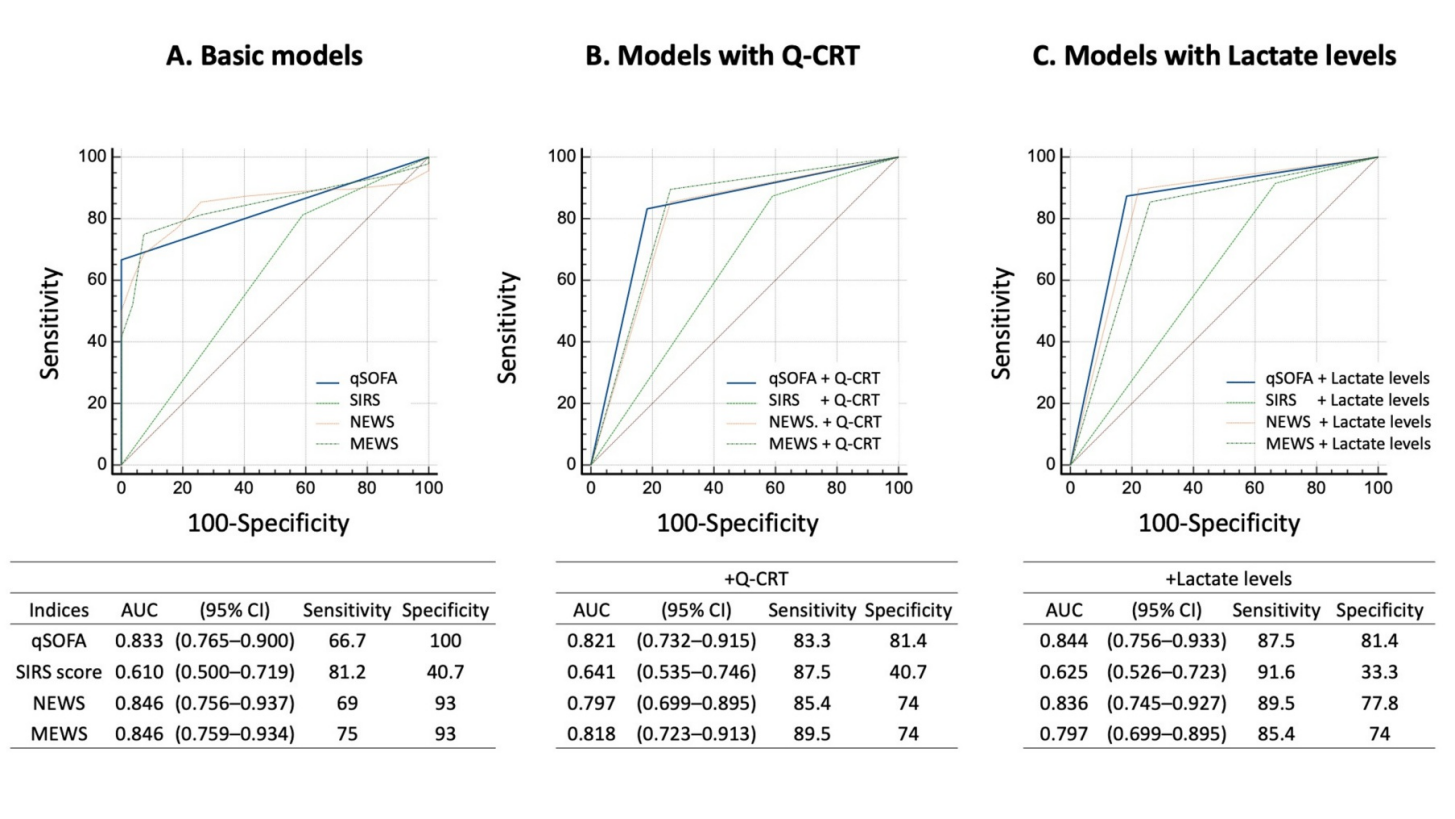
Receiver operating characteristic curves for the screening tools for predicting sepsis A: Predictive ability of the screening tools for sepsis when used alone. B: Predictive ability of each screening tool for sepsis when the Q-CRT was added. C: Predictive ability of each screening tool for sepsis when the lactate level was added AUC: area under the curve; CI: confidence interval; MEWS: Modified Early Warning Score; NEWS: National Early Warning Score; Q-CRT: quantitative capillary refill time; qSOFA: quick Sequential Organ Failure Assessment; SIRS: systemic inflammatory response syndrome

We evaluated the predictive accuracy of eight combinations of screening tools with either Q-CRTs or lactate levels. In combination with Q-CRTs, the qSOFA score showed the highest predictive ability, with an AUC of 0.821, sensitivity of 83.3%, and specificity of 81.4%. Similarly, in combination with lactate levels, the qSOFA score again had the highest predictive ability, with an AUC of 0.844, sensitivity of 87.5%, and specificity of 81.4%. Only the combinations of qSOFA+Q-CRT and qSOFA+lactate levels exceeded 80% in terms of both sensitivity and specificity. Although the qSOFA score, NEWS, and MEWS had high AUCs, they demonstrated low sensitivity. Adding Q-CRTs or lactate levels improved the sensitivity while maintaining the same AUC, compensating for this shortcoming (Figure 3).

## Discussion

In this study, 48 out of 75 cases were diagnosed with sepsis, of which 16 were qSOFA-negative. A review of these cases revealed that many involved pneumonia, where patients remained qSOFA-negative despite SpO₂ desaturation, provided that blood pressure was maintained or that tachypnea was present without altered consciousness. These findings suggest that qSOFA may fail to identify sepsis cases without hypotension when either an altered mental status or an increased respiratory rate is absent, highlighting a potential limitation in its sensitivity for detecting sepsis.

Seymour et al. [7] used a large database to assess the association between each scoring system mentioned in the 2016 Sepsis-3 guidelines and in-hospital mortality in patients with suspected infections outside the ICU. The qSOFA score demonstrated the highest predictive ability for in-hospital mortality, leading to its recommendation as a sepsis screening tool outside the ICU. However, the qSOFA score focuses on the outcomes of patients with sepsis and does not differ according to the cause of the infection [17]. Although the qSOFA can effectively identify patients with poor sepsis outcomes, it cannot be used alone in many cases of sepsis; a combination with other indicators is necessary to address this concern. Moreover, the 2021 SSCG recommends against using only the qSOFA score as a screening tool for sepsis or septic shock and recommends that the screening be performed using the SIRS score, NEWS, or MEWS [11].

The 2021 SSCG suggests measuring blood lactate levels in adults with suspected sepsis. The association between blood lactate levels and mortality in patients with suspected infection or sepsis is well established [18]. This measurement is recommended as part of the SSCG 1-h bundle for patients with sepsis [19], with increased lactate levels included in the Sepsis-3 guidelines for septic shock. Several studies support the use of lactate levels to screen for sepsis when it is clinically suspected but not confirmed [20,21]. Although the qSOFA score is not recommended as a screening tool, it is useful as an indicator of mortality. Therefore, combining the qSOFA score with screening markers, such as Q-CRTs and lactate levels, could be more effective for sepsis screening.

Oi et al. [13] compared the predictive capabilities of the novel Q-CRT with those of the qSOFA and SIRS scores for sepsis screening in emergency departments. The combination of Q-CRTs and qSOFA scores had a higher sensitivity and specificity than the qSOFA or SIRS scores alone. Furthermore, the NEWS and MEWS have been reported to outperform the qSOFA score in screening patients in emergency departments, and a study confirmed that, when used alone, these tools predicted sepsis better than the qSOFA score [22]. Regarding the NEWS, it has been reported that a cutoff value of 8-9 points has excellent diagnostic characteristics for sepsis, with a cutoff value of 7.5 aligning with these findings [23].

In this study, we explored the improvement in the sensitivity of the qSOFA score when it was combined with other screening markers. By adding lactate levels or Q-CRTs to the qSOFA score, its sensitivity was enhanced, with an AUC exceeding 0.8 and sensitivity exceeding 80%, demonstrating that these combinations are superior to the qSOFA score alone for sepsis screening. In contrast, when the SIRS score was combined with Q-CRTs or lactate levels, the AUC remained 0.6, and the specificity was low, ranging from 33% to 40%, revealing the limitations of the SIRS score as a sepsis screening tool. In this study, 55 out of 75 cases were SIRS-positive, of which 16 were not diagnosed with sepsis. Conversely, among the 20 SIRS-negative cases, nine were diagnosed with sepsis. Given that fever is often associated with tachycardia, patients meeting both criteria may fulfill the SIRS definition, potentially leading to a high false-positive rate.

Furthermore, a substantial proportion of SIRS-negative cases were also qSOFA-negative. Notably, cases involving pneumonia with SpO₂ desaturation but preserved blood pressure and normal mental status were frequently missed. These findings suggest that the limitations of SIRS may stem from its susceptibility to false-positive results due to fever-induced tachycardia and its inability to detect sepsis cases lacking hypotension or altered mental status. The decrease in specificity observed when combining SIRS with Q-CRT or lactate can be attributed to the minimal increase in positive cases: only three additional cases with SIRS+Q-CRT and seven with SIRS+lactate. Given that both Q-CRT and lactate are designed to enhance sensitivity rather than specificity, their combination with SIRS likely identified overlapping cases, thereby contributing to the reduced specificity.

Although the NEWS and MEWS alone had higher AUCs than the qSOFA score alone, their sensitivities were lower. Adding Q-CRT and lactate levels improved both sensitivity and specificity, but neither exceeded 80%. The increase in sensitivity and specificity is likely due to Q-CRT and lactate detecting sepsis cases that were not identified by qSOFA. These findings suggest that combining lactate levels or Q-CRT with the qSOFA score is crucial for sepsis screening. This approach is expected to promote the early detection and treatment of sepsis, thereby improving patient outcomes.

Limitations

This study has a few limitations. This was a multi-facility observational study, and hence the results are limited in terms of generalizability. Owing to the limited sample size, the statistical power may be low. Furthermore, the validity of the screening tools we studied should be verified in future prospective studies.

## Conclusions

Our findings show that combining traditional sepsis screening tools with additional markers such as Q-CRT and lactate levels significantly improves the sensitivity and specificity of early sepsis detection. The qSOFA score alone, while valuable as an outcome predictor, lacks sufficient sensitivity for reliable screening. However, its combination with Q-CRT or lactate levels enhances diagnostic accuracy, addressing its limitations. The qSOFA+Q-CRT and qSOFA+lactate combinations both yielded high AUC values and sensitivity and specificity exceeding 80%, making them superior to standalone tools and other combinations in clinical utility. These findings emphasize the need for integrated screening approaches in emergency settings to facilitate timely intervention and reduce sepsis-related mortality. Despite these promising results, the study has several limitations, including its retrospective design and relatively small sample size, which may affect the generalizability of the findings. Future prospective studies with larger cohorts are necessary to validate the effectiveness of these combinations in diverse clinical settings. By improving early detection, this approach has the potential to enhance patient outcomes and provide a robust framework for sepsis management.

## Appendices

**Table 4:** Data from patients with infections Alb: albumin; Bil: bilirubin; BP blood pressure; BT: body temperature; BUN: blood urea nitrogen; Crea: creatinine; Cl: chloride; CRP: C-reactive protein; DBP: diastolic blood pressure; GCS: Glasgow Coma Scale; Hb: hemoglobin; HR: heart rate; Ht: hematocrit; K: potassium; Lac: lactate; MEWS: Modified Early Warning Score; Na: sodium; NEWS: National Early Warning Score; P/F partial pressure of oxygen/fraction of inspired oxygen; Plt: platelets; Q-CRT: quantitative capillary refill time; qSOFA: quick Sequential Organ Failure Assessment; RR: respiratory rate; SBP: systolic blood pressure; SIRS: systemic inflammatory response syndrome; SOFA: Sequential Organ Failure Assessment; SpO_2_: oxygen saturation; TP: total protein; WBC: white blood cells

Patient no.	Sepsis	Q-CRT (sec)	qSOFA	SIRS	NEWS (points)	MEWS (points)	HR (/min)	Lac (mmol/L)	qSOFA+Q-CRT	NEWS+Q-CRT	MEWS+Q-CRT	SIRS+Q-CRT	qSOFA+Lac	NEWS+Lac	MEWS+Lac	SIRS+Lac	Chief complaint	Diagnosis	Death	Fever	Septic shock	SOFA (points)	Age (yrs)	Sex (male)	Height (cm)	Weight (kg)	GCS <15	SBP (mmHg)	DBP (mmHg)	RR (/min)	SpO_2 _(%)	BT (℃)	WBC (/μL)	Hb (g/dL)	Ht (%)	Plt (μL)	TP (g/dL)	Alb (g/dL)	BUN (mg/dL)	Crea (mg/dL)	Bil (mg/dL)	CRP (mg/dL)	Na (mEq/L)	K (mEq/L)	Cl (mEq/L)
4	0	2.009	0	0	4	2	96	1.0	0	0	0	0	0	0	0	0	Sore throat	Streptococcus infection	0	0	0	0	36	1	166	71	0	124	93	28	98	36.8	12600	14.7	43.8	21.5	6.9	4.3	19	0.89	0.8	5.68	140	4.1	105
14	1	2.519	1	1	14	10	144	2.4	1	1	1	1	1	1	1	1	Weakness	Pneumonia	0	1	0	6	56	1	171	68	1	112	62	36	86	40.1	12400	12.1	35.5	6.6	6.5	2.1	54	1.8	1.4	19.6	132	4.5	96
18	1	8.263	0	0	0	1	69	4.2	1	1	1	1	1	1	1	1	Fever	Choledocholith	0	1	0	5	97	0	140	35	0	134	72	18	99	37.7	4900	12.6	36.7	7.3	5.8	3.4	21	0.79	3.1	0.48	141	3.9	104
33	1	3.671	0	1	10	4	80	1.5	1	1	1	1	0	1	0	1	Fever	Podarthritis	0	1	0	2	84	1	159	64	0	152	94	28	88	39.6	9600	11.1	34.4	19.2	6.6	3.3	12	0.76	1.2	17.2	140	2.3	97
39	0	4.114	0	1	4	3	110	1.9	1	1	1	1	1	1	1	1	Fever	Urinary tract infection	0	1	0	0	72	0	155	53	0	132	63	24	98	38.3	13800	10.4	31	16.6	6.7	3.5	15	0.92	1.1	10.9	137	3.7	100
53	1	2.685	0	0	2	2	101	1.8	0	0	0	0	1	1	1	1	Fever	Pneumonia	0	1	0	4	81	1	166	64	0	131	68	16	95	37.4	14300	13.7	42	8.1	6.3	3.5	14	0.82	1.6	3.42	138	3.3	106
54	0	1.981	0	0	2	1	100	1.0	0	0	0	0	0	0	0	0	Fever	Urinary tract infection	0	1	0	1	77	1	155	55	0	119	64	16	94	36.1	22200	11.6	35.5	19.3	6.2	2.9	15	1.33	2.1	11.5	140	3.9	102
67	1	7.255	1	0	6	4	88	1.3	1	1	1	1	1	0	0	0	Consciousness	Hemolytic-uremic syndrome	0	1	1	10	66	1	160	55	1	88	60	16	99	37.3	8800	11.8	34.6	4.3	6.5	2.8	61	4.3	1.9	24.4	134	3.3	97
88	1	3.949	0	1	12	8	140	2.2	1	1	1	1	1	1	1	1	Dyspnea	Pyothorax	0	1	0	4	45	1	170	90	0	114	76	40	91	38.5	38600	14.6	44.1	28.9	6.8	2.8	34	1.75	1.4	46.7	131	4.7	93
111	0	1.578	0	0	5	3	84	1.1	0	0	0	0	0	0	0	0	Facial edema	Erysipelas	0	0	0	1	94	1	158	57	1	105	67	16	95	36.1	8900	13.1	38.8	23.3	6.3	2.7	26	0.83	0.5	8.21	139	4.6	106
112	1	2.693	0	1	7	7	136	1.8	0	0	1	1	1	1	1	1	Fever	Urinary tract infection	0	1	0	2	58	1	173	62	0	177	97	24	96	39.6	12100	12	34.6	18.5	6.9	4.1	11	0.81	2.4	5.32	140	3.9	104
116	1	3.673	0	1	6	3	96	0.9	1	1	1	1	0	0	0	1	Weakness	Pneumonia	0	1	0	2	18	0	155	47	1	124	79	20	95	38.1	8200	13	39.4	18.5	6.6	3.8	17	0.78	0.8	3.18	136	4	98
123	0	6.047	0	1	5	3	106	1.5	1	1	1	1	0	0	0	1	Abdominal pain	Colon perforation	0	1	0	1	67	1	170	55	0	154	87	26	97	38.1	19300	13.7	40.8	46.4	6.5	2.8	24	0.81	0.7	36.8	128	4.7	86
128	0	1.591	0	0	4	1	92	1.2	0	0	0	0	0	0	0	0	Abdominal pain	Appendicitis	0	1	0	1	44	1	180	85	0	146	100	20	93	35.6	18600	16.1	48.5	23.9	7.3	4.6	10	0.87	1.2	19.3	139	4	103
147	0	2.420	0	1	2	3	76	1.5	0	0	0	1	0	0	0	1	Fever	Cellulitis	0	1	0	1	49	1	171	90	0	128	58	18	98	39.1	18800	9.6	28.5	22.4	6.2	2.7	17	1.21	0.6	23.1	135	3.7	101
156	0	3.258	0	0	3	3	75	1.4	0	0	0	0	0	0	0	0	Fever	Enteritis	0	1	0	0	62	1	158	58	0	121	70	30	99	36.5	10700	13.6	39.3	25.5	7	3.3	10	0.88	0.5	12.5	137	3.9	103
167	0	1.415	0	0	3	3	94	0.8	0	0	0	0	0	0	0	0	Fever	Vasculitis	0	1	0	0	67	0	154	61	0	141	81	18	96	39.7	7700	11.7	34.8	17.2	6.8	3.3	10	0.73	0.4	9.61	138	3.2	102
168	0	1.291	0	0	2	3	117	3.0	0	0	0	0	1	1	1	1	Buttock pain	Cellulitis	0	1	0	0	57	1	166	82	0	122	81	20	97	37.2	8600	13.9	41.5	16.6	6.8	3.6	13	0.71	0.9	18.6	141	3.9	103
181	0	1.234	0	1	7	4	126	1.1	0	0	0	1	0	0	0	1	Abdominal pain	Pyothorax	0	0	0	1	65	1	168	72	0	137	96	24	91	37.4	17400	15.3	44.8	45.4	7.1	3.2	19	0.73	0.9	30.6	137	4.6	101
182	1	1.326	1	1	14	10	130	2.6	1	1	1	1	1	1	1	1	Fever	Urinary tract infection	0	1	0	5	72	1	163	60	1	134	74	30	92	40.5	5100	15.9	46.6	11.6	6.8	4	22	1.48	1.7	4.44	140	4	106
184	0	1.105	0	1	7	6	133	1.5	0	0	1	1	0	0	1	1	Vomiting	Urinary tract infection	0	1	0	1	79	0	153	58	0	141	79	35	94	37.8	17500	12.6	36.9	22.1	7.5	3.8	20	0.91	1.3	14.9	139	3.6	104
190	1	6.623	1	1	6	5	105	6.1	1	1	1	1	1	1	1	1	Fever	Necrotizing fasciitis	0	1	1	3	62	1	168	68	0	100	65	33	97	37.1	11100	14.2	42.3	15.8	5.9	3.2	24	2.15	1.9	28.4	129	4	94
204	0	1.825	0	1	6	4	115	1.6	0	0	0	1	0	0	0	1	Diarrhea	Enteritis	0	1	0	0	24	0	160	42	0	115	63	21	95	38.4	19900	13.4	38.7	19.4	6.6	3.9	9	0.79	0.7	15.5	137	4	102
207	1	5.961	1	1	7	5	105	1.9	1	1	1	1	1	1	1	1	Fever	Appendicitis	1	1	0	2	76	1	174	62	1	128	70	25	96	36.4	17500	13.2	38.6	26.9	6.4	2.8	30	0.99	0.8	26.3	135	3.8	98
211	0	1.784	0	1	4	3	108	1.5	0	0	0	1	0	0	0	1	Abdominal pain	Appendicitis	0	1	0	0	38	1	176	89	0	150	88	25	96	38	13900	15.9	47.5	19.5	7	4.5	15	0.93	1.1	3.13	141	3.9	104
215	1	6.799	1	1	11	6	99	1.9	1	1	1	1	1	1	1	1	Weakness	Urinary tract infection	0	1	0	2	77	0	140	55	1	106	73	24	92	39.3	10300	12	34.7	18.4	6.4	3.7	21	0.55	0.8	2.16	137	3.7	103
224	0	3.472	0	0	2	2	82	1.5	0	0	0	0	0	0	0	0	Abdominal pain	Choledocholith	0	0	0	1	74	0	147	49	0	156	104	22	98	36.7	10400	12.8	38.3	21.4	7.1	4.1	14	0.7	1.2	0.04	143	3.4	106
231	0	6.048	0	1	5	4	98	1.0	1	1	1	1	0	0	0	1	Abdominal pain	Enteritis	0	1	0	0	23	1	170	63	0	126	81	24	99	39.6	10100	16	47.8	17.9	6.7	3.9	7	1.05	1	15.8	133	3.6	97
252	0	3.220	0	0	1	1	74	2.4	0	0	0	0	1	1	1	1	Facial edema	Cellulitis	0	0	0	0	66	1	168	86	0	123	91	20	99	35.2	6100	15.1	44.3	20.6	6.8	3.9	13	0.75	0.5	0.19	141	4.2	108
255	0	2.442	0	1	7	5	120	1.6	0	0	1	1	0	0	1	1	Fever	Influenza	0	1	0	1	86	0	140	45	0	154	92	30	99	36.5	10200	13.2	39.8	19.7	5.4	3.1	9	0.49	0.4	2.71	140	4.1	104
265	1	3.898	1	1	12	8	126	1.4	1	1	1	1	1	1	1	1	Fever	Pneumonia	0	1	0	2	84	1	167	50	1	147	96	25	96	39.6	10900	13.5	40.6	19.5	7.1	3.9	24	0.95	0.6	0.64	143	3.9	107
288	1	9.155	1	1	5	3	82	2.6	1	1	1	1	1	1	1	1	Fever	Pneumonia	0	1	0	2	82	0	160	65	1	122	69	19	100	38	26110	15.3	46.3	18.2	6.7	3.2	19.7	0.97	0.9	10.6	140	5.2	103
290	0	2.915	0	1	2	3	100	1.1	0	0	0	1	0	0	0	1	Weakness	Choledocholith	0	1	0	1	78	1	160	50	0	128	63	20	96	38.6	9890	8.9	26.7	18.8	6	2.2	19.2	0.75	1.2	20	135	2.5	100
293	1	5.059	1	1	17	13	149	4.3	1	1	1	1	1	1	1	1	Fever	Pneumonia	1	1	1	7	65	1	175	45	1	66	37	30	94	39.9	600	9.6	28.1	11.1	5.1	2.9	22.9	1.18	0.5	8.5	136	3.3	108
294	1	2.697	0	0	0	1	78	1.3	0	0	0	0	0	0	0	0	Abdominal pain	Enteritis	0	0	0	2	44	0	155	50	0	135	81	18	98	36.4	23090	13.5	40.3	28.3	7	4.3	7.6	0.58	2.4	7.3	140	3.6	107
295	1	1.947	1	1	9	5	107	1.8	1	1	1	1	1	1	1	1	Fever	Pneumonia	0	1	0	2	79	0	150	40	1	121	50	27	98	37.5	6000	12	37.6	21.9	6.2	3.1	14.3	0.65	0.4	3.2	140	4.4	105
296	1	5.580	1	1	11	8	122	2.7	1	1	1	1	1	1	1	1	Fever	Cholecystitis	0	1	1	8	81	0	150	35	1	103	78	30	99	37.5	11550	11.2	32.9	22.1	6.1	3.1	97.7	2.19	1.8	10.6	156	3.9	111
297	1	9.403	1	1	9	7	126	7.7	1	1	1	1	1	1	1	1	Fever	Urinary tract infection	0	1	0	2	42	1	170	60	1	124	62	28	100	38.1	16960	15.3	43.7	42.7	8.2	4.6	17.4	0.94	1.1	0.7	138	4	99
298	0	2.207	0	0	1	1	74	1.0	0	0	0	0	0	0	0	0	Abdominal pain	Diverticulitis	0	0	0	0	69	0	158	45	0	105	56	20	98	37.2	4570	12.9	40.2	24.3	6.9	3.5	7.1	0.65	1.1	13	138	3.9	105
303	1	7.094	1	1	11	5	102	1.8	1	1	1	1	1	1	1	1	Dyspnea	Pneumonia	0	1	0	2	83	1	165	55	1	182	102	27	93	36.8	10480	13.7	41.8	33.1	7.2	2.9	26.8	0.37	0.3	9.3	149	2.7	110
305	1	5.868	0	1	9	4	110	3.4	1	1	1	1	1	1	1	1	Dyspnea	Pneumonia	0	0	0	2	86	0	140	47	0	150	81	32	86	37.1	21070	12.8	37.9	21.7	7.4	3.8	17.6	0.74	1	5.1	140	4.1	107
308	1	6.438	1	0	8	5	83	2.7	1	1	1	1	1	1	1	1	Dyspnea	Pneumonia	0	0	0	3	83	1	166	68	1	138	63	36	96	36.6	11910	14.3	43.6	12.2	5.5	3.1	30.3	0.85	1.1	13.7	145	4.5	108
309	1	0.908	1	1	9	6	100	1.2	1	1	1	1	1	1	1	1	Fever	Urinary tract infection	0	1	0	4	86	0	150	40	1	140	69	23	99	38.9	13830	14.1	41.1	9.2	7.3	4.3	18.1	0.53	1.6	1.9	138	2.9	102
311	1	11.535	1	1	13	6	122	1.5	1	1	1	1	1	1	1	1	Dyspnea	Pneumonia	1	0	0	3	81	0	142	40	1	142	62	7	90	36.8	27400	10	31.6	28.6	5.5	1.9	27.5	1.15	0.3	18.9	145	3.6	107
313	1	2.137	0	1	7	3	115	1.7	0	0	0	1	1	1	1	1	Fever	Enteritis	0	1	0	7	68	1	165	55	0	95	83	13	90	37.7	10400	13.8	40.5	4.1	6.2	2.5	45.9	1.51	2.5	22.4	132	4.3	98
314	1	3.143	0	0	1	0	94	0.9	0	0	0	0	0	0	0	0	Fever	Enteritis	0	0	0	5	43	1	165	63	0	123	79	14	97	36.3	2160	15	41.3	5.1	6.5	3.4	9.4	0.99	2.9	16.1	136	3.7	102
315	1	1.881	1	1	8	6	114	1.3	1	1	1	1	1	1	1	1	Fever	Pneumonia	0	1	0	2	84	1	158	58	1	160	76	24	94	37.9	17020	13.4	39.7	13.3	6.5	4	17.4	0.93	0.8	5.8	136	4	102
316	1	4.589	1	1	11	5	107	2.9	1	1	1	1	1	1	1	1	Dyspnea	Pneumonia	0	1	0	4	90	1	155	50	1	124	67	24	82	38	15070	11.7	37.1	18.8	5.3	2.9	19.5	0.73	1.2	1.1	144	4.1	109
317	0	4.385	0	1	4	3	102	4.2	1	1	1	1	1	1	1	1	Dyspnea	Pneumonia	0	0	0	0	66	1	175	52	0	147	108	28	99	37.2	13950	15.1	43.4	40.9	6.9	3	8.9	0.77	0.6	13	130	4.2	96
318	1	5.194	1	1	13	8	94	2.1	1	1	1	1	1	1	1	1	Fever	Urinary tract infection	0	1	0	3	88	0	138	40	1	96	64	30	93	39.6	10570	9	30	22.4	6.9	3	31.3	1.6	0.4	4.3	144	5.2	111
319	1	3.568	1	1	15	10	183	4.0	1	1	1	1	1	1	1	1	Fever	Cholangitis	0	1	1	7	86	0	150	40	1	96	66	27	96	39.2	9730	11.8	35.3	13.4	7.4	3.5	33.4	1.36	4.3	18.8	139	4.3	108
320	0	2.000	0	1	4	3	85	1.0	0	0	0	1	0	0	0	1	Fever	Urinary tract infection	0	1	0	1	70	0	151	35.2	1	146	101	16	98	38.4	13680	13.6	42.2	31.6	7.8	4.4	15.4	0.55	0.5	7.1	142	3.9	100
321	1	2.474	1	1	12	7	134	1.9	1	1	1	1	1	1	1	1	Fever	Pneumonia	0	1	0	3	84	1	170	65	1	123	83	22	95	38.1	11350	9.9	31	21.3	6.5	3.1	34.3	1.8	0.4	2.7	142	4.4	113
322	1	4.297	1	1	17	8	150	14.8	1	1	1	1	1	1	1	1	Dyspnea	Pneumonia	0	1	1	9	78	1	170	55	1	82	48	28	81	37.3	11540	12.4	39.5	34.8	6.2	1.7	30.3	1.2	1	29.5	144	4.9	110
325	1	11.018	1	1	13	8	110	3.1	1	1	1	1	1	1	1	1	Fever	Pneumonia	1	1	0	4	81	1	160	50	1	162	74	34	80	38.9	10960	11.4	34.3	20.5	6.1	2.7	18.4	0.96	0.9	19.1	140	3.3	102
326	1	11.051	0	1	9	5	112	1.9	1	1	1	1	1	1	1	1	Fever	Pneumonia	0	0	0	2	59	1	175	63	0	130	81	38	94	35.8	8270	16.8	45.6	16.4	6.7	2.8	56.7	1.88	1.1	40.1	129	3.3	88
328	0	2.912	0	0	5	2	87	1.4	0	0	0	0	0	0	0	0	Fever	Bronchitis	0	0	0	1	77	1	162	52	0	120	86	26	96	37.8	9620	13.9	39.9	16.6	6.7	3.4	23.4	0.9	1.7	20.7	135	4.2	101
329	1	1.706	0	0	8	3	86	1.2	0	1	0	0	0	1	0	0	Dyspnea	Pneumonia	0	0	0	4	86	0	150	42	1	151	78	15	82	37.9	5950	11.5	35.4	10.4	10.8	1.7	54.7	1.65	0.4	5.8	130	4	100
330	1	2.800	1	1	12	8	101	1.2	1	1	1	1	1	1	1	1	Fever	Pneumonia	1	1	0	2	79	1	175	60	1	114	69	31	95	39.7	12820	11.9	35.1	37.3	6.4	2.4	12	0.49	0.6	20.1	131	4.3	100
332	1	6.765	1	1	17	11	145	5.6	1	1	1	1	1	1	1	1	Dyspnea	Pneumonia	1	0	1	10	59	1	165	75	1	240	140	32	84	36.4	16040	16.3	48	23.4	7.3	4.5	13.9	1.16	0.4	0.7	136	4.9	102
333	0	1.525	0	1	9	3	105	2.1	0	1	0	1	1	1	1	1	Dyspnea	Pneumonia	1	1	0	1	70	1	165	71	0	132	83	25	92	38.4	12430	12.3	37.4	26.1	6.4	3.2	13.6	0.68	0.4	12.7	137	4.8	103
335	1	1.322	1	0	8	5	109	2.7	1	1	1	0	1	1	1	1	Fever	Urinary tract infection	1	1	0	5	82	0	155	50	1	85	60	16	94	37.4	8520	11.3	34.2	5.4	4.9	2.1	25.3	1.09	0.6	13.2	140	3.5	109
336	1	2.581	1	1	14	8	96	2.6	1	1	1	1	1	1	1	1	Fever	Pneumonia	0	1	0	3	92	1	160	55	1	87	55	30	99	39.2	8020	7.8	24.4	19.7	5.4	2.3	26.6	0.94	0.6	22.7	134	3.6	102
338	1	2.465	0	1	6	5	148	7.1	0	0	1	1	1	1	1	1	Omalgia	Cholangitis	1	0	0	7	79	1	159	78	0	104	54	22	97	36.7	6560	8.1	25.4	2.7	6.1	2	72.3	2.03	4.6	14.5	130	5.8	100
339	1	1.790	1	1	11	9	137	3.1	1	1	1	1	1	1	1	1	Consciousness	Meningitis	1	1	0	5	63	1	164	55	1	176	82	28	98	40	10800	11.5	36.1	10.5	8.4	2.7	15.2	0.71	2.4	3.8	126	4.7	98
341	1	6.221	1	1	12	7	105	0.8	1	1	1	1	1	1	1	1	Dyspnea	Pneumonia	1	0	0	9	88	1	162	60	1	134	84	30	86	36.8	6680	8.6	28	17.4	5.3	2.4	55.5	4.04	0.7	9.8	149	4.2	115
342	1	2.295	1	1	11	7	117	9.4	1	1	1	1	1	1	1	1	Consciousness	Pneumonia	0	0	0	4	93	0	145	35	1	174	109	23	93	36.5	9490	14.1	42.3	22	7.2	4	14.1	0.67	0.4	0.3	126	4.1	91
343	0	3.269	0	1	8	4	94	1.4	0	1	0	1	0	1	0	1	Fever	Pneumonia	0	1	0	1	94	1	165	57	0	147	66	26	94	39	6050	12	39.2	18.4	7.8	3.9	28.9	1.82	0.6	6.2	142	4	111
347	1	5.648	1	1	11	6	110	1.9	1	1	1	1	1	1	1	1	Dyspnea	Nontuberculous mycobacterial infection	0	1	0	2	76	1	161	69	1	168	108	36	95	38.1	12170	13.9	41.8	22.4	8.2	3.2	26.9	0.74	0.5	36.2	135	3.8	97
348	1	2.955	0	0	7	5	114	1.9	0	0	1	0	1	1	1	1	Fever	Pneumonia	0	1	0	7	75	1	163	57	1	104	70	16	95	37.5	8670	14.3	43.5	11.7	6.1	2.9	73.8	5.39	0.8	41.7	140	6.2	107
350	0	4.249	0	1	6	4	91	1.2	1	1	1	1	0	0	0	1	Fever	Pneumonia	0	1	0	0	86	1	165	50	0	122	59	25	94	38.7	12510	9.6	33.6	31.5	5.5	3	16.4	0.73	0.2	0.6	142	4.1	113
352	1	8.176	0	1	1	3	77	1.2	1	1	1	1	0	0	0	1	Fever	Urinary tract infection	0	1	0	2	85	1	160	48	0	146	81	15	98	38.8	12700	14.2	42	13.5	6.4	3.2	15.5	1.11	1.2	7.7	138	4.2	102
354	1	9.234	0	1	4	5	66	2.4	1	1	1	1	1	1	1	1	Fever	Pneumonia	1	1	0	0	81	1	168	67	1	153	67	15	97	38.6	12760	9.1	27	27.6	4.5	1.7	18.7	1.28	1.1	1.6	134	3.9	102
355	1	2.539	1	1	11	10	182	3.2	1	1	1	1	1	1	1	1	Fever	Legionella pneumonia	1	1	1	14	91	0	147	45	1	150	114	18	95	39.3	11440	16.5	51.7	5.2	4.8	2.9	92.7	3.47	2.1	1	147	5.6	108
357	0	2.024	0	1	4	3	78	1.1	0	0	0	1	0	0	0	1	Fever	Urinary tract infection	0	1	0	1	78	1	163	73	1	140	74	20	96	38.3	19100	13	38.3	18.1	6.8	3.7	15.6	0.94	0.6	19.4	138	3.7	104
